# Right Coronary Artery to Right Atrial Fistula: Role of Multi-Modality Imaging and Percutaneous Closure

**DOI:** 10.7759/cureus.26716

**Published:** 2022-07-10

**Authors:** Ahmad Jabri, Zaid Shahrori, M. Farhan Nasser, Keith Bullinger, Anas Alameh, Faris Haddadin, Ahmad Al-Abdouh

**Affiliations:** 1 Heart and Vascular Center, MetroHealth Medical Center/Case Western Reserve University, Cleveland, USA; 2 Medicine, Hashemite University, Zarqa, JOR; 3 Vascular Medicine, Cleveland Clinic, Cleveland, USA; 4 Internal Medicine, MetroHealth Medical Center, Cleveland, USA; 5 Internal Medicine, Cleveland Clinic Akron General, Akron, USA; 6 Cardiology, Baylor College of Medicine, Houston, USA; 7 Internal Medicine, Saint Agnes Hospital, Baltimore, USA

**Keywords:** right atrium, percutaneous closure, cardiac magnetic resonance imaging, atrial fibrillation, coronary artery fistula

## Abstract

Coronary artery fistula (CAF) is a connection between a coronary artery and a cardiac chamber or nearby vessel. Our case represents a fistula arising from the right coronary artery and terminating in the right atrium, presenting as atrial fibrillation. CAF closure options include surgical and percutaneous approaches.

## Introduction

Coronary artery fistula (CAF) is defined as a connection arising between a coronary artery and a cardiac chamber or another nearby vessel. CAF can be congenital, iatrogenic, or acquired secondary to trauma. Pacemaker implantation, endomyocardial biopsy, and coronary angiography are considered invasive cardiac procedures in which CAF may arise [1]. In most cases, CAFs appear as an isolated finding (90.5%); however, CAF can be associated with other congenital cardiac malformations, including atrial septal defects, patent foramen ovale, pulmonary artery atresia, tetralogy of Fallot, ventricular septal defects, and patent ductus arteriosus [2]. We present a case of a patient who presented with atrial fibrillation and, on further investigation, was found to have a CAF. The patient was treated using the percutaneous approach.

## Case presentation

History of presentation

A 60-year-old male patient with a past medical history of type 2 diabetes mellitus was referred to the cardiology clinic for dyspnea of six months duration and a new diagnosis of atrial fibrillation (AF). Due to worsening symptoms, a transesophageal echocardiogram (TEE)-guided cardioversion was planned to attempt restoration of normal sinus rhythm (NSR).

Differential diagnosis

Differential diagnoses included aortic diverticulum, aortic dissection pouch, Kawasaki disease, and a coronary artery fistula.

Investigation

**Figure 1:**
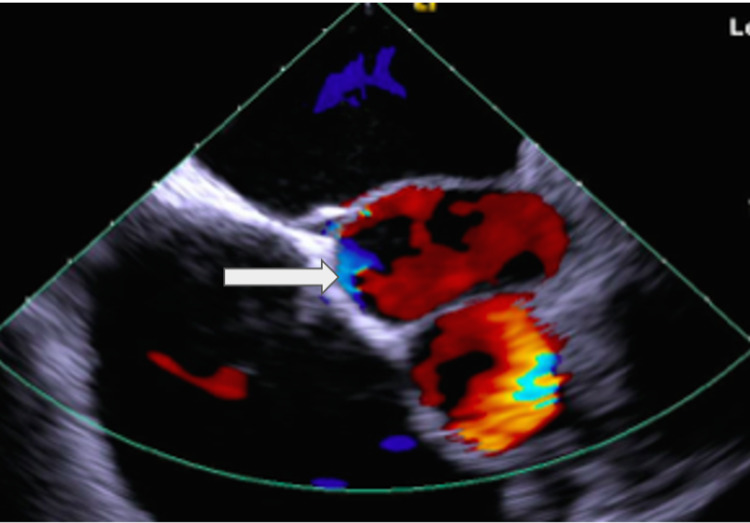
A transesophageal echocardiogram (mid-esophageal short-axis view) shows an abnormal structure (4 x 4 cm) adjacent to the aorta (white arrow).

The patient underwent TEE, which revealed normal left ventricular systolic and right ventricular systolic functions. Atria was mildly enlarged. The left atrial (LA) appendage was free of thrombus. The most striking abnormality was an abnormal vascular structure of 4 x 4 cm adjacent to the ascending aorta (Figure 1).

**Figure 2:**
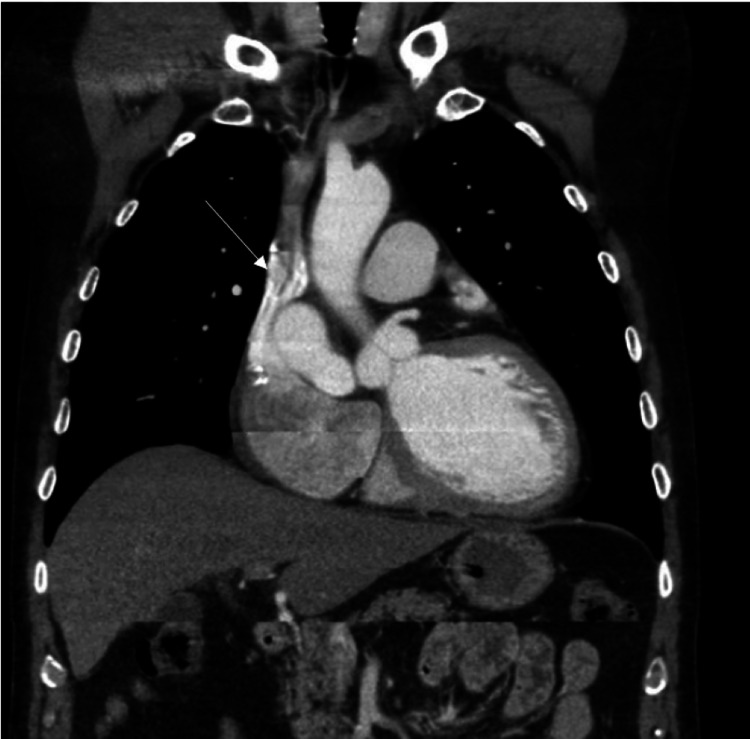
Chest CT showing saccular fistula (white arrow) measuring 3.6 x 3.3 cm connecting the right coronary artery to the right atrium.

**Figure 3:**
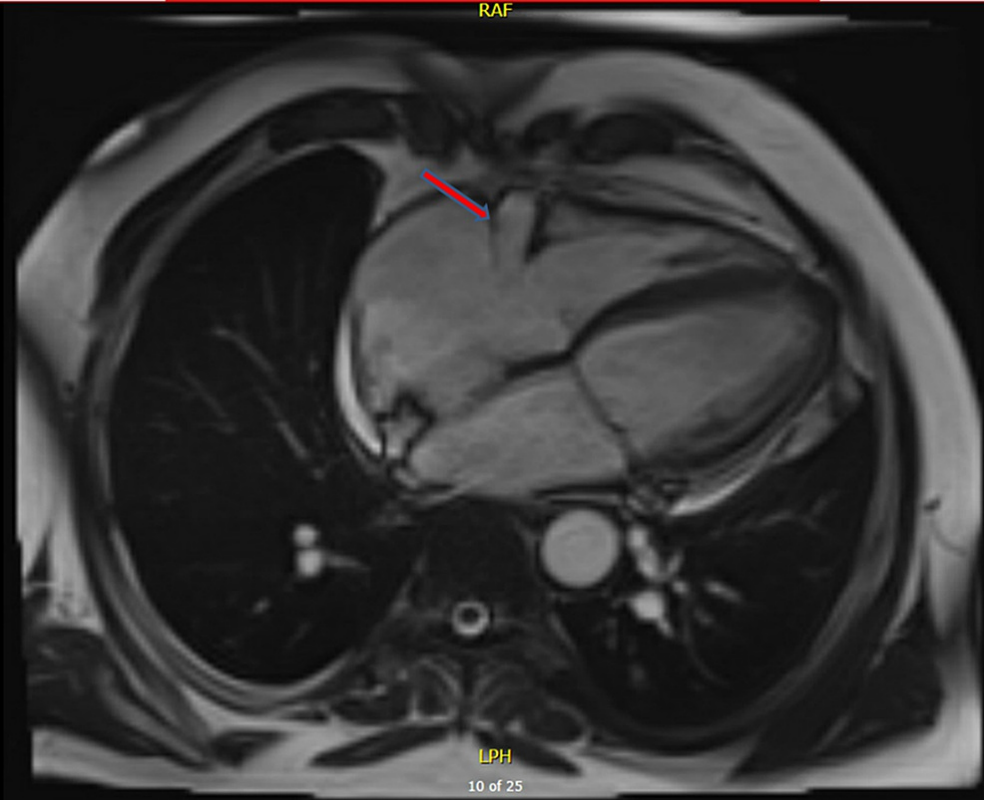
Cardiac MRI showing the fistula and shunting (red arrow).

**Figure 4:**
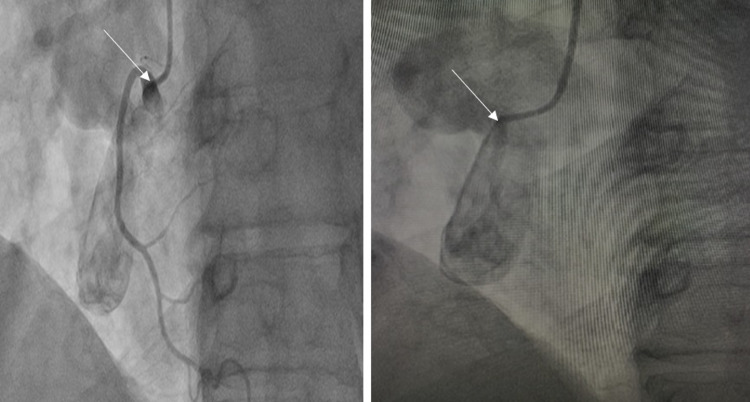
Fistula arising from the ostium of the right coronary artery and emptying into the right atrium (white arrow).

The patient was cardioverted to NSR with 200 joules of synchronized shock. Chest computed tomography (CT) was performed next for further evaluation. It revealed a large, very tortuous, and saccular fistula measuring 3.6 x 3.3 cm connecting the right coronary artery (RCA) to the right atrium (RA) (Figure 2). A cardiac MRI was also performed for further assessment confirming the presence of fistula and significant shunting (Figure 3). The shunt fraction of pulmonary blood flow to systemic blood flow (Qp:Qs) was 1.7, indicating a significant left-to-right shunt. Afterward, coronary angiography was performed, which revealed a large fistula arising from the ostium of the RCA and emptying into the RA (Figure 4). A significant amount of contrast media passed from the aorta into the RA through the fistulous connection.

Management

**Figure 5:**
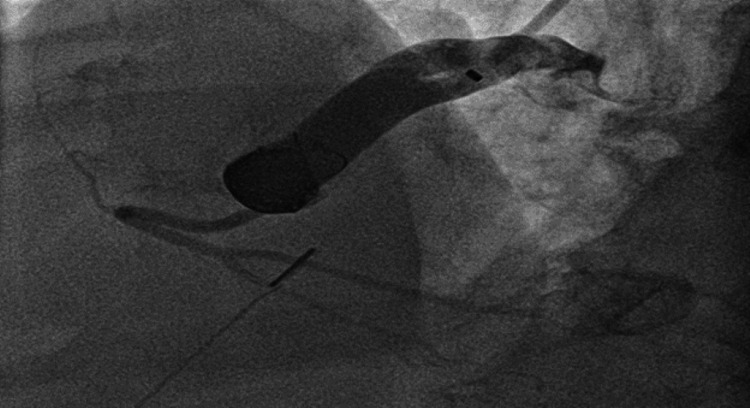
The patient undergoing percutaneous closure with a 16 mm Amplatzer Vascular Plug II and coils.

The structural heart team was consulted to evaluate percutaneous therapeutic options instead of more invasive surgical options. The patient later underwent percutaneous closure with a 16 mm Amplatzer Vascular Plug II (Abbott Laboratories, Chicago, IL) and coils (Figure 5). Angiography following the procedure showed occlusion of the fistula. The patient was discharged on apixaban, aspirin, and clopidogrel with a plan to discontinue clopidogrel after one month and continue aspirin and apixaban indefinitely. The patient reported improvement in symptoms in the one-month follow-up visit.

## Discussion

The most common site of origination of CAFs is the RCA with an incidence of 33%, followed by the left coronary artery (LCA) (34.9%), left anterior descending artery (6.3%), circumflex artery (4.8%), and finally, both RCA and LCA (1.6%) [3]. CAFs do not only arise from different coronaries but also terminate in different cardiac chambers and nearby vessels. The right ventricle (RV) is the most prevalent drainage site for CAFs, making up 34.9% of cases. The RA (27%) and pulmonary artery (PA) (27%) are the second most common sites of drainage and are followed by the left ventricle (LV) (6.3%), coronary sinus (CS) (3.2%), and finally LA (1.6%) [3].

The clinical manifestations of CAFs rely on the diameter of the fistula and the difference between the systemic blood pressure affecting the coronary artery and the blood pressure in the draining chamber. In most circumstances, CAFs are relatively small, and patients are asymptomatic. However, CAFs can result in the coronary artery steal phenomenon and deviate blood flow through the coronary arteries into the terminating chambers. Symptoms may arise because of oxygen supply deprivation to the myocardium. Heart failure, myocardial infarction, pulmonary hypertension, endocarditis, arrhythmias, thrombosis of the fistula, or fistula rupture may also occur. A loud continuous murmur at the lower sternal or midsternal border is the most prevalent finding of CAFs. Few cases have been reported in the literature of CAFs presenting with atrial fibrillation. Two previously reported atrial fibrillation cases were secondary to CAF arising from the RCA and ended in either the superior vena cava or the RA. The location of the fistula likely contributed to the evolution of AF in those scenarios and our case [4].

The diagnosis of CAF can be challenging. Initial evaluation of the patient should involve an electrocardiogram, which could be normal, show chamber enlargement, arrhythmias, or signs of ischemia, depending on the site and flow of the fistula. Two-dimensional and color Doppler transthoracic echocardiogram (TTE) or TEE are valuable tools for evaluating patients with CAF [5]. Moreover, cardiac MRI will allow identification of the site, size, and shunt flow. A cardiac CT angiogram is another modality for diagnosis. However, coronary angiography is still the gold standard of diagnosis.

CAF management is mainly related to the presence or absence of symptoms and the amount of shunting. Symptomatic patients can be candidates for either surgical or percutaneous closure. On the other hand, the management of asymptomatic CAFs varies depending on the risk of complication, for which high risk will require treatment versus lower risk may only require monitoring [6]. The course, tortuosity, and the coexistence of aneurysmal dilation dictate the approach used in the fistula closure. Patients with CAF who require a bypass graft or surgical valve repair/replacement should undergo surgical closure.

Moreover, CAFs with high flow, branching course, tortuosity, or coexistent aneurysmal enlargement are better approached with the surgical technique. Patients with CAFs who did not meet the above characteristics can better tolerate percutaneous closure [7]. Transcatheter closure is preferred when applicable due to the lower risk of bleeding, infections, wound healing, general anesthesia adverse effects, and cardiopulmonary bypass complications. Post-closure medical therapy recommendations include antiplatelet medications and prophylactic antibiotics for infective endocarditis for the first six months before undergoing high-risk procedures [8].

Complications of CAF include recurrence rates of 9-19% with transcatheter closure versus 25% with surgical ligation [9]. Other complications include myocardial infarction, tricuspid regurgitation, thromboembolism, or death. Post repair, the recorded survival rates of coronary artery fistulas in the literature were 93%, 74%, and 68% at one, five, and 15 years, respectively [10]. In the case presented by Jamali et al., the author mentioned complete resolution of atrial fibrillation post obliteration of the CAF [4].

## Conclusions

CAFs have a broad spectrum of presentations. The characteristics of the fistula dictate the severity of signs and symptoms and will later influence the treatment approach. Arrhythmias, including AF, can be the presenting feature of CAFs and may be eradicated post obliteration of the CAF. Percutaneous and surgical approaches have been associated with recurrence, and therefore long-term follow-up is more appropriate.
